# Mother–infant interactions and regional brain volumes in infancy: an MRI study

**DOI:** 10.1007/s00429-016-1347-1

**Published:** 2016-12-03

**Authors:** Vaheshta Sethna, Inês Pote, Siying Wang, Maria Gudbrandsen, Anna Blasi, Caroline McCusker, Eileen Daly, Emily Perry, Kerrie P. H. Adams, Maria Kuklisova-Murgasova, Paula Busuulwa, Sarah Lloyd-Fox, Lynne Murray, Mark H. Johnson, Steven C. R. Williams, Declan G. M. Murphy, Michael C. Craig, Grainne M. McAlonan

**Affiliations:** 1 Department of Forensic and Neurodevelopmental Sciences, Sackler Institute for Translational Neurodevelopment, Institute of Psychiatry, Psychology and Neuroscience, King’s College London, PO 50, 16 De Crespigny Park, London, SE5 8A UK; 2 Department of Engineering Science, Institute of Biomedical Engineering, University of Oxford, Oxford, UK; 3 Centre for Brain and Cognitive Development, Birkbeck, University of London, London, UK; 4 Division of Imaging Sciences and Biomedical Engineering, Centre for the Developing Brain, King’s College London, London, UK; 5 School of Psychology and Clinical Language Sciences, University of Reading, Reading, UK; 6 Stellenbosch University, Stellenbosch, South Africa; 7 Department of Neuroimaging, Institute of Psychiatry, Psychology and Neuroscience, King’s College London, London, UK; 8 NIHR Biomedical Research Centre for Mental Health at the South London and Maudsley NHS Foundation Trust and King’s College London, London, UK; 9 GKT School of Medical Education, King’s College London, London, UK

**Keywords:** Mother–infant interaction, Infant brain structure, MRI, Infancy, Sex differences, Maternal sensitivity, Infant cerebellum

## Abstract

It is generally agreed that the human brain is responsive to environmental influences, and that the male brain may be particularly sensitive to early adversity. However, this is largely based on retrospective studies of older children and adolescents exposed to extreme environments in childhood. Less is understood about how normative variations in parent–child interactions are associated with the development of the infant brain in typical settings. To address this, we used magnetic resonance imaging to investigate the relationship between observational measures of mother–infant interactions and regional brain volumes in a community sample of 3- to 6-month-old infants (*N* = 39). In addition, we examined whether this relationship differed in male and female infants. We found that lower maternal sensitivity was correlated with smaller subcortical grey matter volumes in the whole sample, and that this was similar in both sexes. However, male infants who showed greater levels of positive communication and engagement during early interactions had smaller cerebellar volumes. These preliminary findings suggest that variations in mother–infant interaction dimensions are associated with differences in infant brain development. Although the study is cross-sectional and causation cannot be inferred, the findings reveal a dynamic interaction between brain and environment that may be important when considering interventions to optimize infant outcomes.

## Introduction

Parent–infant interactions are critical for child development. For instance, sensitive and responsive early care is linked to optimal behavioural and cognitive outcomes (Cabrera et al. 2011; Lugo-Gil and Tamis-LeMonda 2008); in contrast, parental insensitivity increases the risk of children developing psychopathology in later life (Murray et al. 2010). Although the biological mechanisms mediating these associations are not entirely understood, it is generally agreed that the human brain is most vulnerable to environmental influences (De Bellis et al. 2001; Schore 2001)—including parent–infant interactions (Rifkin-Graboi, et al. 2015)—early in development.

For example, elevated levels of stress hormones stemming from early-life adversity are thought to lead to altered brain development through the accelerated loss of neurons, disrupted pruning, inhibition of neurogenesis (Teicher et al. 2006; Tupler and De Bellis 2006), and perhaps also altered anatomical ‘connectivity’ (Sarkar et al. 2014). Prior reports also suggest that early childhood maltreatment is associated with later fronto-limbic abnormalities (Belsky and de Haan 2011; Hart and Rubia 2012); smaller corpus callosum and total brain volumes, and increased ventricular volumes (De Bellis et al. 2002; Teicher et al. 2004). It has also been suggested that the male brain is particularly vulnerable to such insults (De Bellis and Keshavan 2003; Tupler and De Bellis 2006). For example, smaller cerebral volumes have been reported in older male children exposed to childhood maltreatment (Belsky and de Haan 2011).

However, existing studies in humans mainly document outcomes following extreme adversity in infancy (i.e., institutional rearing or severe maltreatment), and are retrospective in design. Also, the high prevalence of psychopathology (72%) in these retrospective analyses of older cohorts (De Bellis et al. 2001) makes it difficult to determine whether the structural brain differences observed explain the aetiology of psychopathology or are caused by it and/or its treatment [for example, medication exposure may confound interpretation (Tupler and De Bellis 2006)]. Thus, the results of these high-risk samples do not reveal how normative variations in early parent–child interactions influence child brain structure in the early postnatal period.

This is an important omission, considering the compelling evidence that an early sensitive caregiving environment likely provides an optimal emotional context for children’s early brain maturation and subsequent cognitive abilities (Bernier et al. 2010). Furthermore, the postnatal period is characterized by rapid brain development. Specifically, the first year of life is the period of greatest brain volume growth in typical children—total brain volume at 2–4 weeks of age is approximately 36% of adult volume, and by 1 year it is approximately 72% of adult volume (Knickmeyer et al. 2008). Brain plasticity during this period makes the infant brain particularly sensitive to environmental influence, especially the social-affective environment (Schore 2001). Variations in maternal care are thought to help shape neural structures and circuits, and subsequently psychological outcomes (Roth and Sweatt 2011); and there is reasonable consensus that maternal sensitivity in the first year of life has a key impact on development (de Wolff and van Ijzendoorn 1997). Defined as the timely and accurate response to the infant’s communicative cues, maternal sensitivity predicts positive social relationships and enhanced cognitive abilities in the infant (Wade et al. 2015); and sensitive caregiving during the first year is critical for the maturation of the infant’s stress response system (Gunnar and Cheatham 2003; Hane and Fox 2006).

Therefore, the relationship between normative variations in parenting and brain structure in children has now started to be examined (Kok et al. 2015; Moutsiana et al. 2015; Rao et al. 2010; Rifkin-Graboi, et al. 2015; Whittle et al. 2014). For example, higher levels of parental sensitivity in early childhood have been linked with larger total brain and grey matter volumes in children at 8 years of age (Kok, et al. 2015). In another study, insecure attachment at 18 months was associated with greater amygdala volumes at 22 years (Moutsiana et al. 2015). In contrast, a study of structural MRI data from twenty 6-month-old infants, has demonstrated a link between maternal sensitivity and hippocampus volume (Rifkin-Graboi et al. 2015)—specifically, reduced maternal sensitivity was associated with larger volumes. While these studies are important first steps, some had a lengthy period between caregiving measures and brain MRI acquisition (Kok et al. 2015; Moutsiana et al. 2015), and others used adolescent samples (Whittle et al. 2014). In infancy and childhood, the changes in brain volume over time occur in parallel to maturation of cognitive, motor and socio-emotional processes (Shulman 2016; van Soelen et al. 2012). By examining the brain and the factors that influence it at the same time, we can begin to identify possible causes of altered brain growth and behaviour, as well as potential treatment targets and biomarkers that are predictive of outcomes.

In the current study, we used magnetic resonance imaging (MRI) to investigate whether mother–infant interactions observed in a community sample of mothers and their 3- to 6-month-old infants, are related to variations in regional brain volumes. In addition to studying an association between maternal behaviours and infant brain volume, it is important to know whether—or not—infant behaviours are related to brain volumes as this may help us understand what brain systems drive infant behaviour and/or respond to infant behaviour changes. Such information may eventually help us develop objective predictive tools to identify infants who might benefit from early intervention to improve outcomes. Moreover, since there is a bidirectional link between maternal sensitivity and infant behaviours (Beebe et al. 2016; Feldman 2007; MacLean et al. 2014), it is also possible that infant behaviours relate to brain development indices. Therefore, we predicted that there would be a relationship between both maternal and infant behaviours and regional brain volumes. Owing to limited prior information in infancy, with both larger and smaller regional brain volumes reported in relation to early caregiving, and no previous evidence in relation to infant behaviours, the direction of this relationship was not a priori predicted.

Finally, where an association between brain regions and mother–infant interactions was observed, we conducted an exploratory examination of potential sex differences in these relationships. As preclinical and clinical studies of adverse rearing conditions (i.e., exposure to childhood maltreatment) indicate that the male brain is influenced more by the early environment (Belsky and de Haan 2011; Glaser 2000), we predicted that any relationship between maternal and/or infant behaviour and brain would be stronger in males.

## Methods

### Participants

Participants were 43 mother–infant dyads recruited from the local community in London. The aim was to capture data from infants aged around 4 months. For logistical reasons, infants were eligible for the study if they were aged between 3 and 6 months, born at term (gestational age >36 weeks) with no congenital abnormalities. Mothers had to have a working knowledge of the English language, and be free of any current or past major psychiatric illness, or any antenatal or obstetric complications potentially altering infant development (for example, perinatal asphyxia). Exclusion criteria included contraindications for MRI scanning (for example, metallic implants or pacemakers). Written informed consent was obtained from mothers for the protocol approved by the UK National Research Ethics Committee (REC 08/H0718/76, 06/MRE02/73 and 12/LO/2017).

A total of four MRI scans were excluded from the analysis due to poor image quality driven by motion artefacts (*n* = 3), and an incidental brain anatomical anomaly (*n* = 1). Hence, the final sample included 39 infants (mean age = 4.83 months, SD = 1.15 months; 51.3% male) with data on both measures, i.e., mother–infant interactions and brain volumes from MRI scans. Of the total sample, 51.3% (*n* = 20) were male, and there was no difference in infant age at scan between the sexes (*p* = 0.577). Maternal and infant demographic characteristics for the total sample, and split by infant sex, are presented in Table 1.

**Table 1:** Maternal and infant demographic characteristics for the whole sample and by infant sex

	Whole sample (*N* = 39)	Males (*n* = 20)	Females (*n* = 19)	Sex difference statistic (*p* value)
Infant demographics				
Age at MRI (months); mean (SD)	4.83 (1.15)	4.73 (1.20)	4.94 (1.11)	*t* = −0.563, *p* = 0.577
Gestational age at birth (weeks); mean (SD)	39.71 (1.95)	39.85 (1.83)	39.57 (2.11)	*t* = −0.445, *p* = 0.659
Birth weight (g); mean (SD)	3390.51 (527.48)	3490.50 (424.05)	3285.26 (612.19)	*t* = −1.22, *p* = 0.229
Weight at MRI (g); mean (SD)	7105.30 (1302.95)	7262.51 (1172.48)	6939.81 (1440.83)	*t* = −0.77, *p* = 0.450
Maternal demographics				
Age (years); mean (SD)	33.82 (4.45)	33.90 (4.48)	33.74 (4.53)	*t* = −1.13, *p* = 0.911
Ethnicity; *n* (%)				*χ* ^2^ = 4.47, *p* = 0.214
White	32 (82.1)	18 (90.0)	14 (73.7)	
Asian	4 (10.3)	1 (5.0)	3 (15.8)	
Black	1 (2.6)	1 (5.0)	0 (0.0)	
Mixed race	2 (5.1)	0 (0.0)	2 (10.5)	
Educational level; *n* (%)				χ^2^ = 1.25, *p* = 0.536
GCSE and A-levels	2 (5.1)	1 (5.0)	1 (5.3)	
Vocational college	4 (10.3)	1 (5.0)	3 (15.8)	
Higher education	33 (84.6)	18 (90.0)	15 (78.9)	

*SD* standard deviation, *GCSE* General Certificate of Secondary Education, *A-Levels* General Certificate of Education Advanced Level, *Higher education* undergraduate and postgraduate degree

### Procedures

#### Mother–infant interactions

Observations of mother–infant interactions were video-recorded for 5 min using a standard assessment protocol of face-to-face play (Murray et al. 1996b)—with the infant placed in an infant seat. Mothers were instructed to play with and talk to their infant as they normally would, but without using any toys or objects. Maternal and infant behaviours were coded by two trained raters using the Global Rating Scales (GRS, Murray et al. 1996b), which are sensitive to impaired interactions even in low-risk samples (Gunning et al. 2004).

The first five uninterrupted continuous play minutes of videotaped mother–infant interaction were coded as in previous studies (Halligan et al. 2013). Maternal communication modalities coded were sensitivity and affect. These dimensions were included since maternal sensitivity is known to predict infant and child cognitive outcomes, and high levels of negative affectivity disrupt the infant’s regulatory capacity and quality of parent–infant relationships, leading to maladaptive child outcomes (Murray et al. 1996a; Murray and Trevarthen 1986). Additionally, two infant dimensions were included—communication and affective state—both of which are critical for shaping cognitive outcomes (Cates et al. 2012).

In line with previous work, the dimensions were scored on a standard five-point scale, where 1 corresponds to “poor” interactive maternal or infant behaviour and 5 to most “optimal” behaviour. Dimensions of mother–infant interactions were derived as per standard use in previous studies (Murray et al. 1996a; Stein et al. 2012).

1. *Sensitivity* Maternal response to the infant’s communication cues; the extent to which it is contingent and appropriate to the infant’s needs and experiences; also including attitude and feelings towards the infant. Maternal sensitivity was characterized by warmth, acceptance, non-demanding, and non-intrusive behavioural dimensions.
2. *Affect* Maternal demonstration of affective state, including positive and negative affectivity (i.e., depressive-like expressions). Affective state was characterized by level of maternal enjoyment, effort and vitality, degree of self-consciousness, and the extent of anxiety in the interaction.
3. *Communication* Infant’s level of engagement and communication (i.e., positive vocal and non-vocal behaviour directed towards the mother). Communication included the amount of visual contact, and positive vocalizations, in addition to other forms of communication (for example, mouthing, movement of limbs).
4. *Fretfulness* Infant’s affective state and level of distress.

Inter-rater intraclass correlations (Shrout and Fleiss 1979) were measured on a randomly selected 20% of the interactions, and ranged from 0.741 to 0.993, indicating good-to-excellent inter-rater reliability. Discrepancies between raters were discussed, and final ratings were determined in collaboration with members of the Winnicott Research Unit who were involved in the development of the scale. ICCs stated for the GRS scales utilized absolute scores and were calculated prior to adjustments to ratings—that is, they include the original values by raters.

#### MRI data acquisition

MRI data were acquired on a 1.5-T General Electric scanner (GE Medical Systems, Milwaukee, WI, USA), equipped with an 8-channel head coil. Infants were scanned in natural sleep; further details can be found in Blasi et al. (2011). A T2-weighted fast spin echo (T2w) sequence with the following imaging parameters was acquired: number of slices = 20; slice thickness = 4 mm; slice gap = 2 mm; repetition time = 3000/4500 ms; echo time = 115 ms; field of view = 180 mm; flip angle = 90°; matrix size = 256 × 224. The structural sequence used for this study was necessarily a short scan acquired alongside functional MRI. All images were analysed blind to mother–infant interaction ratings.

#### Image processing and volumetric segmentation

The T2w MR images were first skull-stripped using label propagation and decision fusion of three manual brain masks (Heckemann et al. 2006). Segmentations of the masked images were then performed using an atlas-based method, which adapted the Statistical Parametric Mapping (SPM v.8) software, and a probabilistic neonatal brain atlas (Kuklisova-Murgasova et al. 2011) as an input to the SPM software. The SPM segmentation model unifies tissue classification, image bias correction and non-linear atlas registration (Ashburner and Friston 2005). Iterated Conditional Modes were employed to optimize the Gaussian mixture model (GMM) parameters for the tissue intensity distributions, the bias field parameters and the atlas deformation parameters. The GMM parameters were estimated using an expectation–maximization algorithm (Fombonne 2009) and a Levenberg–Marquardt algorithm (Courchesne et al. 2000), to obtain the bias field and deformation parameters. Subsequently, the segmentation of cerebrospinal fluid (CSF) was refined by thresholding the masked T2w image based on the mean of the intensity distribution calculated using the SPM posterior probability map of CSF. The partial volume misclassifications by this intensity-based SPM segmentation model were corrected using second order Markov random fields, which enabled spatial constraints to be imposed by configuring a three-dimensional connectivity tensor (Erskine et al. 2013). Following this automated protocol, one rater examined all images in a final manual editing process using ITK-SNAP (v.2.2) (Yushkevich et al. 2006).

**Fig. 1:**
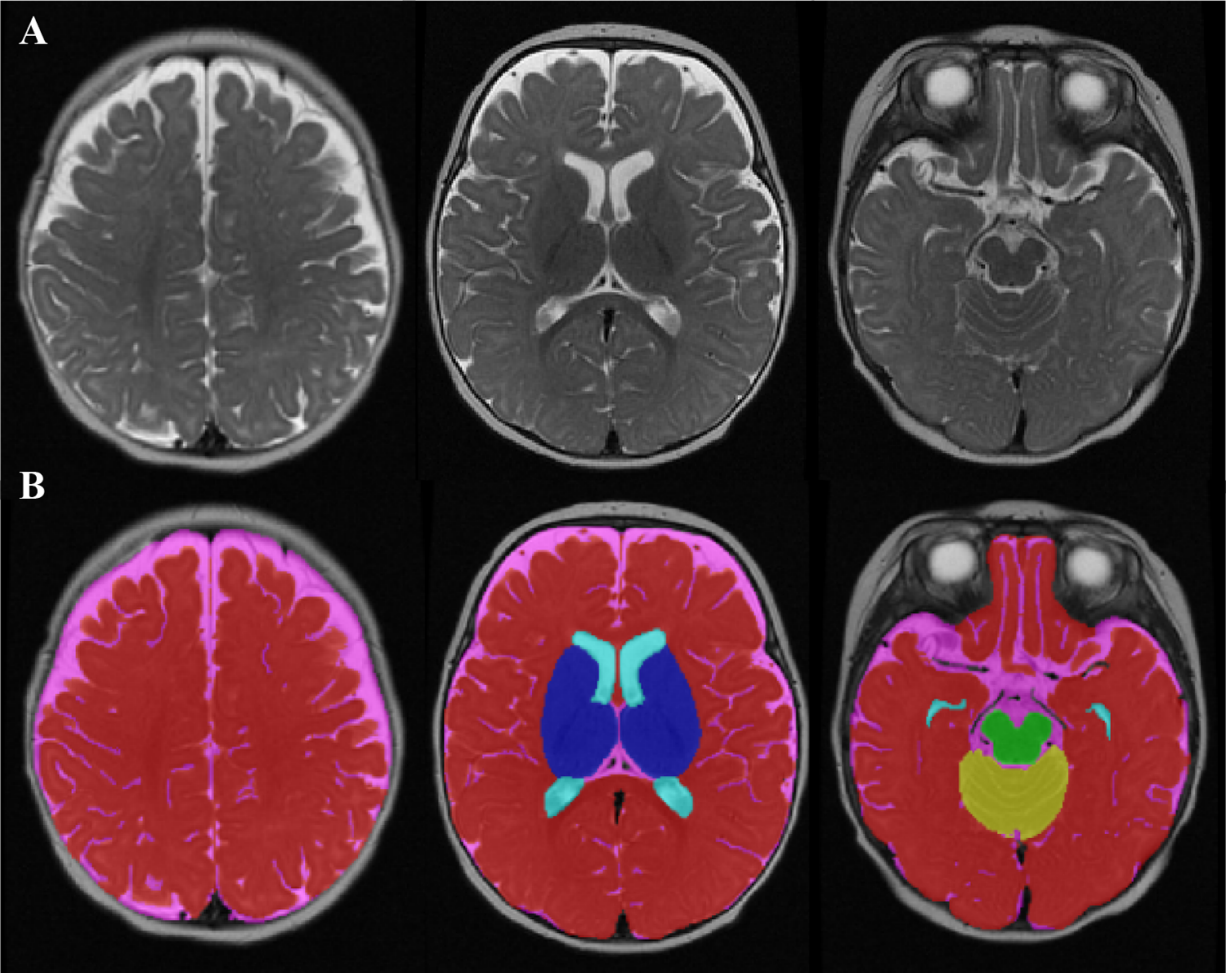
Volumetric segmentation of a 4-month-old brain. **a** T2-weighted axial MRI image of a 4-month-old infant brain. **b** The final result of the volumetric segmentation, with label maps for CSF (*pink*), lateral ventricles (*light blue*), midbrain (*green*), cerebellum (*yellow*), subcortical grey matter (*dark blue*), and total grey and white matter (*red*)

This process yielded volumes of the following brain regions (Fig. 1): (a) CSF (including both CSF, third ventricle and fourth ventricle); (b) lateral ventricles (including the cavum septum pellucidum and vergae); (c) midbrain (including the cerebral peduncle, substantia nigra, brainstem and pons); (d) cerebellum; (e) subcortical grey matter (including the caudate, putamen, globus pallidus and thalamus), and the remaining (f) total grey and white matter. Further grey and white matter segmentation was not conducted given the difficulty in accurately classifying these tissue classes at this age (Hazlett et al. 2012). Finally, a measure of (g) intracranial volume was also obtained by summing all regions (a–f). All regional brain volumes were expressed as proportions of intracranial volume, and these ‘corrected’ measures were used in the analyses.

#### Volumetric segmentations: intra-rater reliability

The reliability of the volumetric segmentations was confirmed by intra-rater intraclass correlations between the final segmentations, and a repeat measurement of a random 20% selection of the automatically segmented images. For the intracranial volume, the intraclass correlation of the intra-rater variability was 0.998 (*p* < 0.001), indicating excellent reproducibility. Similar results were found for the individual correlations of each brain region: CSF (0.989, *p* < 0.001), lateral ventricles (0.965, *p* < 0.001), midbrain (0.918, *p* < 0.001), cerebellum (0.948, *p* < 0.001), subcortical grey matter (0.923, *p* < 0.001), and total grey and white matter (0.984, *p* < 0.001). The ICCs stated were derived from absolute measurements.

### Statistical analysis

Data were analysed using the IBM SPSS (Statistical Package for the Social Sciences) Software Package (v.22) (SPSS Chicago, IL, USA). We examined the relationships between mother–infant interactions and brain volumes across the entire cohort; when these were present we then examined the relationship in male and female infants separately.

First, descriptive data were examined to confirm that these conformed to assumptions of normality. Regional brain volume measures were ‘corrected’ (expressed as proportions of intracranial volume) and the sexes compared. Next, a set of planned bivariate correlation analyses between the maternal and infant interaction indices and brain volumes was calculated. A threshold of at least a moderate effect size (*r* > 0.3) with the significance level set to *p* < 0.05 was selected as preliminary evidence for a relationship between behaviour and brain volume (Cohen 2013; Kotrlik and Williams 2003). Second, after a Bonferroni correction for multiple correlations, where a significant association emerged, the association between interaction dimension and brain volume was further examined using the PROCESS macro tool (Hayes 2013). We estimated whether the interaction term between each mother and infant behaviour and sex (i.e., maternal interaction dimension × sex and infant behaviour × sex) was associated with brain structure volume. Infant age and weight at time of scan were included as covariates, to adjust for age and weight differences in brain volume (Parikh et al. 2013). We also controlled for maternal education (an index of socio-economic status), which has been linked to brain structure (Brito and Noble 2014). PROCESS applies bias-corrected bootstrapping intervals to probe the interaction term and make inferences about indirect effects, rather than relying on the normality assumption. The number of bootstrap samples used to determine 95% bias-corrected bootstrap confidence intervals was 10,000. PROCESS also produces the conditional effects of the independent variable at the two values of a binary moderator (sex: male = 0, female = 1).

## Results

Table 2 shows the means, standard deviations, and sex differences for mother–infant interaction dimensions and brain volumes. Significant sex differences were found in the raw measures of the subcortical grey and intracranial volumes; in both instances, male infants had larger volumes than females—subcortical grey: (males: mean = 36.17, SD = 4.16; females: mean = 33.05, SD = 2.36; *t* (37) = 2.89, *p* = 0.007), and intracranial volume (males: mean = 888.56, SD = 88.54; females: mean = 825.20, SD = 102.68; *t* (37) = 2.07, *p* = 0.046).

**Table 2:** Mother–infant interaction dimensions and brain volumes: whole group descriptive statistics and comparisons by infant sex

	Whole sample (*N* = 39)	Males (*n* = 20)	Females (*n* = 19)	Sex difference	
	Mean (SD)	Mean (SD)	Mean (SD)	*t*	*p* value
Interaction dimensions^a^					
Maternal dimensions					
Sensitivity	3.48 (0.54)	3.51 (0.52)	3.45 (0.56)	−0.30	0.765
Affect	4.25 (0.53)	4.26 (0.54)	4.23 (0.52)	−0.21	0.836
Infant dimensions^a^					
Communication	3.52 (0.91)	3.76 (0.86)	3.28 (0.91)	−1.70	0.098
Fretfulness	4.11 (0.69)	4.06 (0.66)	4.15 (0.74)	0.40	0.695
Brain volumes, cm^3^					
Total grey and white matter	586.12 (69.66)	603.53 (69.35)	567.80 (66.92)	1.64	0.110
Midbrain	13.90 (1.91)	14.42 (1.83)	13.35 (1.89)	1.79	0.082
Subcortical grey	34.65 (3.71)	36.17 (4.16)	33.05 (2.36)	2.89	0.007
Cerebellum	74.83 (11.91)	77.11 (12.51)	72.43 (11.06)	1.24	0.224
Lateral ventricles	14.27 (4.58)	15.01 (4.54)	13.49 (4.62)	1.03	0.308
Cerebrospinal fluid	133.94 (40.70)	142.36 (44.95)	125.07 (34.68)	1.34	0.189
Intracranium	857.71 (99.72)	888.58 (88.54)	825.20 (102.68)	2.07	0.046

^a^Low scores indicate poor interactions (for example, lower levels of sensitivity, increased depressive affect, fewer communication attempts and increased infant fretfulness)

### Relationship between mother–infant interaction dimensions and brain volumes

#### Maternal affect

Maternal affect was positively correlated with total grey and white matter volume, and negatively correlated with CSF volume (*r* = 0.33, *p* = 0.042; *r* = −0.33, *p* = 0.039, respectively). Thus, infants exposed to negative affect (i.e., depressive-like expressions) had smaller total grey and white matter volumes, and larger CSF volumes. However, these associations did not survive correction for multiple testing.

#### Maternal sensitivity

Furthermore, a positive association of moderate effect size was found between maternal sensitivity and subcortical grey matter volume (*r* = 0.54, *p* < 0.001) in the whole sample—i.e., infants interacting with less sensitive mothers had smaller subcortical grey volumes. The association survived correction for multiple comparisons (Bonferroni corrected *p* value =0.001). When adjusting for covariates (infant age, weight and maternal education) the association remained statistically significant (*B* = 0.002, *p* = 0.046), and there was no evidence that infant sex moderated the association between maternal sensitivity and subcortical grey matter volume, as the interaction term (maternal sensitivity × sex) was not significant (*p* = 0.806). Furthermore, none of the covariates were associated with the outcome (i.e., subcortical volume) in the model tested.

#### Infant communication

There was a significant negative correlation of moderate effect size between infant communication and cerebellar volume (*r* = −0.48, *p* = 0.002) in the whole sample—i.e., greater infant communication and engagement during mother–infant interactions was associated with smaller cerebellum volumes. This association also survived correction for multiple comparisons (Bonferroni corrected *p* value =0.020), and remained significant when adjusting for covariates (*B* = −0.01, *p* = 0.003). Covariates associated with the outcome (i.e., cerebellum volume) in the model tested included infant age (*p* < 0.001) and sex (*p* = 0.019)—implying larger cerebellum, in older, male infants. Furthermore, the interaction term (infant communication × sex) in this model was significant (*B* = 0.01, *p* = 0.017), indicating that infant sex moderated the association between infant communication and cerebellum volume (*R* ^2^ increase due to the interaction = 0.09, *F* = 6.32, *p* = 0.017). While the conditional association between infant communication and cerebellum volume was significant in male infants (*B* = −0.05, *p* = 0.003), suggesting smaller cerebellar volumes with increased communication; there was no such evidence in female infants (*B* = 0.00, *p* = 0.762).

#### Infant fretfulness

Infant fretfulness was not significantly correlated with regional brain volumes

## Discussion

In this cross-sectional exploratory study, we show that variations in typical mother–infant interactions are associated with differences in infant brain volumes. Specifically, we found that lower maternal sensitivity was correlated with smaller subcortical grey matter volumes in both sexes. In contrast, male infants with higher levels of communication during early interactions had smaller cerebellar volumes.

Prior studies of extreme neglect, leading to paediatric post-traumatic stress disorder, have reported that childhood maltreatment is associated with smaller total grey and white matter volumes, and larger frontal lobe CSF volumes, especially in males (De Bellis and Keshavan 2003; De Bellis et al. 2002). A more recent investigation of normal variations in parental care and brain structure (at 8 years of age) has revealed a similar relationship between early childhood parental sensitivity and total brain and grey matter volumes (Kok et al. 2015). More specifically, and when compared to other brain regions, the subcortical grey matter appears to be particularly ‘responsive’ to early environmental influences. For example, the basal ganglia and thalami (which comprise the subcortical grey) are very sensitive to hypoxic events in utero (Okereafor et al. 2008; du Plessis and Volpe 2002; Shalak and Perlman 2004); and infants so exposed, tend to have poor neurodevelopmental outcomes.

Our work extends these findings to show that a relationship between maternal sensitivity and infant brain development is present from as early as 3 months. However, these findings are correlational and do not necessarily indicate a causative link between early care and infant brain structure. Also, we cannot say firmly whether this relationship has ‘positive’ or ‘negative’ developmental implications. Neither can we be certain whether smaller regional brain volumes are a consequence of poorer parenting quality, or whether infants with smaller regional brain volumes influence their mothers’ interactions. It is also possible that since infant and mother are closely genetically related, the associations observed could be mediated through shared genetic variants, including an inherited brain volume and behavioural style.

We do suggest, however, that the infant stress response system, which undergoes rapid development in the first year of life, is likely to be involved. For example, in the early postnatal period when the hypothalamic–pituitary–adrenal (HPA) axis of infants is labile, sensitive parenting is associated with either smaller increases or less prolonged activations of the infant HPA axis, when subjected to mild stress (Albers et al. 2008). Therefore, exposure to negative (for example, insensitive or intrusive) parental behaviours may constitute a source of stress for the infant, and activate the infant’s adrenocortical axis (Atkinson et al. 2013). The subsequent elevation in cortisol may influence brain volume and ‘connectivity’ in the growing child (Sarkar et al. 2014). Furthermore, mothers who are more sensitive in the postnatal period have been reported to demonstrate secure mental representations of attachment during pregnancy, which in turn may impact upon the HPA axis and the intra-uterine environment (Kinsella and Monk 2009). Hence, associations between maternal behaviours and infant brain volume may have their origins even earlier in development, but future studies including objective measures of the HPA axis and a comprehensive characterization of maternal psychopathology during pregnancy are needed to better understand the mechanisms involved. In addition, as maternal sensitivity is thought to be a stable trait over time (Feldman 2010), follow-up of these dyads would help to determine whether the relationship we observed between maternal sensitivity and the infant brain persists or shifts as children grow.

An important aspect of our study design was that it also permitted examination of a possible link between infant behaviour (for example, communication and fretfulness) and brain volume. We found that a smaller cerebellum volume is associated with better infant communication, and this relationship was particularly evident in male infants. Again, we emphasize that the causal direction of this relationship is not known—i.e., does cerebellar development drive communication, or vice versa? Regardless, a link between cerebellar development and communication is not surprising given its key role in emotion processing and executive functioning (Schmahmann et al. 2007). For example, the cerebellum has been proposed to have a key role in the temporal processing of events and in allocating attentional resources in ‘real-time’ to guide or prepare behaviour (Schwartze and Kotz 2016). In addition, the cerebellum responds to auditory stimulation including spoken language (Buckner 2013). Together, these attributes likely make a key contribution to organizing effective communication during face-to-face interaction, and our data suggest that the link between cerebellum and communication is present from early infancy. Furthermore, that our results reveal a relationship primarily in males might also have been expected, as the developmental trajectory of the cerebellum is sexually dimorphic. The male cerebellum develops more slowly than the female (Tiemeier et al. 2010), potentially making the former more vulnerable to early adverse environments. Consistent with this, cerebellar pathology is a hallmark of neurodevelopmental disorders, such as ASD, which also shows marked sex differences (Wang et al. 2014). Finally, our sample size and current study design precludes an in-depth analysis of potential processes which might explain the link between infant behaviour and brain volume; including, for example, the role of maternal sensitivity which could be considered in future research.

Our study has a number of limitations. First, as noted above, our results are correlational and causality cannot be inferred. Second, although in line with the current literature (Rifkin-Graboi et al. 2015), our sample size was modest and replication in larger samples will be necessary. Third, the infants in our study were mainly from white European ‘middle-class’ families, educated to degree level, and therefore, we cannot be certain that these results generalize to families of different ethnicities and educational backgrounds. Fourth, we did not define a priori regions of interest since we do not yet have extensive knowledge of all brain areas affected by normative variations of parenting in infancy. Fifth, our primary goal in this initial study was to establish if there were brain regions linked to mother-infant interactions across the group, and then, having done that, to explore if there were sex differences in those specific regions. This approach helped us avoid type 1 error when running multiple tests. However, it risked generating type 2 errors of incorrectly retaining a false-negative finding. Therefore, we cannot exclude the possibility that there are sex differences in regions without main effects; and in our ongoing studies we are recruiting much larger cohorts in order to look at each sex separately across multiple brain regions. We hope this will provide adequate power to explore regional associations with sex and parent–child interactions in detail. Finally, there were also technical constraints to our study. The scanning of very young infants is challenging and the structural sequences used were of relatively low resolution. Hence, our overall volumetric measurement may miss the fine-grained structural differences that might be detectable in larger samples, or through higher resolution scanning protocols. Furthermore, and in line with previous studies of this age range (Hazlett et al. 2012), another limitation was the inability to differentiate between grey and white matter volume, due to ongoing myelination in these young infants.

Nonetheless, the current analyses provide a Proof of Principle that early mother–infant interactions are associated with variations in infant brain development. If correct, our finding that early sensitivity (a modifiable factor) is linked to the development of brain regions (known to impact upon emotional and cognitive development), opens up the potential to influence infant developmental trajectories.
